# Electrocardiographic Changes Related to Targeted Temperature Management in Brugada Syndrome: A Case Report

**DOI:** 10.5811/cpcem.52845

**Published:** 2026-04-29

**Authors:** Yuki Kondo, Atsuhito Tanaka, Tomoya Okazaki

**Affiliations:** * Tokyo Bay Urayasu Ichikawa Medical Center, Department of Emergency and Critical Care Medicine, Urayasu, Chiba, Japan; † Japan Community Health Care Organization Osaka Hospital, Department of Anesthesia and Critical Care Medicine, Fukushima-ku, Osaka, Japan; ‡ Chibanishi General Hospital, Intensive Care Unit, Matsudo, Chiba, Japan

**Keywords:** Brugada syndrome, cardiac arrest, targeted temperature management, intensive care medicine, case report

## Abstract

**Introduction:**

Brugada syndrome is an important differential diagnosis for unexplained sudden cardiac arrest, particularly in younger patients. The electrocardiographic (ECG) pattern characteristic of Brugada syndrome can be provoked by fever and may vary with changes in body temperature. Therefore, targeted temperature management following cardiac arrest may obscure the distinctive morphology, increasing the risk of misdiagnosis.

**Case Report:**

We report the case of a 44-year-old man who experienced out-of-hospital cardiac arrest due to ventricular fibrillation following influenza B infection. Initial evaluation revealed transient ST-segment elevation in leads V1–V3, while coronary angiography and echocardiographic findings were normal. Although Brugada syndrome was suspected, the diagnosis was deferred because the ECG findings normalized during targeted temperature management at 36 °Celsius. However, after completion of temperature management, the patient developed a high-grade fever, accompanied by the emergence of a characteristic coved-type Brugada ECG pattern. Subsequent genetic testing identified a sodium channel protein type 5 subunit alpha mutation return, confirming Brugada syndrome.

**Conclusion:**

Brugada ECG morphology can be affected by core temperature, and repeat electrocardiography during febrile episodes may be informative.

## INTRODUCTION

Brugada syndrome is a hereditary disorder that increases the risk of sudden cardiac arrest in young adults, with structurally normal hearts and preserved cardiac function.^1^–^3^ Accurate diagnosis is crucial to prevent recurrent life-threatening arrhythmias in patients and to assess familial risk, enabling primary prevention. Diagnosis relies on a thorough medial history, family history, and a 12-lead electrocardiogram (ECG) showing a pseudo-right bundle branch block and ST-segment elevation in leads V1–V2. These ECG features may be absent initially and can be influenced by body temperature.^4^ Thus, patient temperature must be taken into account when assessing for Brugada syndrome.

Targeted temperature management is a standard intervention following cardiac arrest.^5^,^6^ Although the optimal target temperature remains unclear, strict fever prevention is widely recommended. This may obscure the Brugada ECG pattern in those cases where the pattern arises when the patient is febrile. We report a case of dynamic ECG changes associated with temperature fluctuations in a patient who experienced sudden cardiac arrest after fever due to influenza infection, leading to the diagnosis of Brugada syndrome.

## CASE REPORT

A 44-year-old male was referred to our hospital due to out-of-hospital cardiac arrest. Four years prior, ECG and coronary computed tomography angiography revealed a right bundle branch block and myocardial hypertrophy. He took no medications on a regular basis. The day before presentation, he was diagnosed with influenza B infection and prescribed oseltamivir. He had no allergies and no family history of sudden death or cardiac disease. Emergency medical services noted the initial rhythm to be ventricular fibrillation. Return of spontaneous circulation was achieved with 1 mg of epinephrine and on the second defibrillation.

On admission to the emergency department, he was hemodynamically stable and did not require vasopressors. His vital signs were as follows: heart rate, 97 beats per minute; blood pressure, 154/93 millimeters of mercury; and temperature, 38.2 °C. He remained unconscious and required intubation for airway protection. His ECG showed ST-segment elevation in V1–V3. See Image (a). Echocardiography demonstrated anterior wall hypokinesis without valvular abnormality, and coronary angiography was normal. Brugada arrhythmia was considered; however, the findings were insufficient for a definitive diagnosis, and takotsubo syndrome was initially suspected. Computed tomography of the head, chest and pelvis showed no notable abnormal findings, and laboratory tests were unremarkable.

Because he was unable to follow commands, targeted temperature management at 36.0 °C was performed for 72 hours using an intravascular cooling device, Thermoguard XP (Zoll Medical Corporation, Chelmsford, MA). Throughout this time, ECG and echocardiography showed no significant abnormalities. Brugada syndrome remained a consideration due to his age and prior ST-segment elevation in V1–V3, but findings were inconclusive. During targeted temperature management, his ECG demonstrated normal sinus rhythm. See Image (b). After temperature management, he developed a temperature of 39.2 °C, and his ECG revealed a definitive Brugada pattern with coved-type ST-segment elevations—Image (c).

*CPC-EM Capsule*

What do we already know about this clinical entity?

*Brugada syndrome is an arrhythmogenic disorder causing sudden cardiac arrest, with electrocardiogram patterns that can fluctuate and be exacerbated by fever.*

What makes this presentation of disease reportable?

*This case shows disappearance of a fever-induced Brugada ECG pattern during targeted temperature management that reappeared during recurrent fever.*

What is the major learning point?

*Brugada ECG patterns may normalize during targeted temperature management; repeat ECG after recurrent fever is essential for diagnosis.*

How might this improve emergency medicine practice?

*Awareness that targeted temperature management can alter Brugada ECG patterns may prompt repeat ECG evaluation and reduce misdiagnosis.*

The patient was suspected to have suffered a malignant arrhythmia secondary to Brugada syndrome triggered by fever resulting from influenza. Genetic testing was later performed to guide family screening and confirmed Brugada syndrome with a sodium channel protein type 5 subunit alpha (SCN5A) mutation.

## DISCUSSION

In this case the key finding was the presence of dynamic ECG changes associated with temperature fluctuations in a patient who experienced sudden cardiac arrest after fever due to influenza infection, leading to the diagnosis of Brugada syndrome. Along with sodium channel-blocking drugs, electrolyte disturbances, bradycardia, vagal stimulation, and certain psychotropic or recreational drugs, fever is recognized as one of the principal provoking factors for arrhythmias in Brugada syndrome and is considered particularly important because it can acutely predispose affected patients to sudden cardiac arrest.^2^,^7^ While previous reports have described fever-induced arrhythmias and ECG features, our case uniquely demonstrates normalization of a fever-provoked Brugada ECG pattern during targeted temperature management, followed by reinduction with recurrent fever. To our knowledge, this is the first report to describe a Brugada ECG pattern induced by influenza-associated fever that varied in response to subsequent changes in body temperature.

An observational study reported that Brugada syndrome was most often recorded at mean temperatures of 39 °C (range 38.4–40 °C).^8^ This phenomenon is attributed to the fact that elevation of body temperature exacerbates dysfunction of cardiac sodium channels, particularly the voltage-gated sodium channel type 1.5 channel associated with SCN5A gene mutations, resulting in a further reduction in the sodium current.^2^ In light of these mechanisms, it is not surprising that, as observed in our case, lowering body temperature led to normalization of the characteristic ECG pattern. Targeted temperature management is one of the standard treatments for patients after cardiac arrest, and it is recommended to maintain body temperature below 37.7 °C during the first 72 hours.^6^ Clinicians should recognize that Brugada patterns may normalize during targeted temperature management.

Even in the absence of diagnostic ECG findings, repeated testing after targeted temperature management is recommended when Brugada syndrome is considered a possible cause of sudden cardiac arrest. Diagnosis of Brugada syndrome is important for both determining the indication for implantable cardioverter-defibrillator therapy in patients at high risk of cardiac arrest and enabling appropriate screening of family members. Survivors of cardiac arrest from Brugada arrhythmia are candidates for an implantable cardiac defibrillator. Furthermore, given the autosomal dominant genetic pattern, genetic counseling and mutation-specific genetic testing are recommended for first-degree relatives of patients with Brugada syndrome.^9^ In this case, positive genetic testing enabled appropriate counseling for family members. These findings underscore the importance of repeated ECG evaluation during the peri-temperature management period in patients with unexplained cardiac arrest.

## CONCLUSION

We present a rare case of an adult male in sudden cardiac arrest due to Brugada arrhythmia induced by fever from influenza B. The characteristic ECG pattern was only revealed after the patient was allowed to spike a fever. Repeated ECG evaluation during the peri-targeted temperature management period enabled diagnosis. We recommend repeating the ECG while monitoring changes in body temperature during that period in patients with unexplained cardiac arrest, particularly in younger adults, to improve recognition of Brugada syndrome.

## Figures and Tables

**Image.**
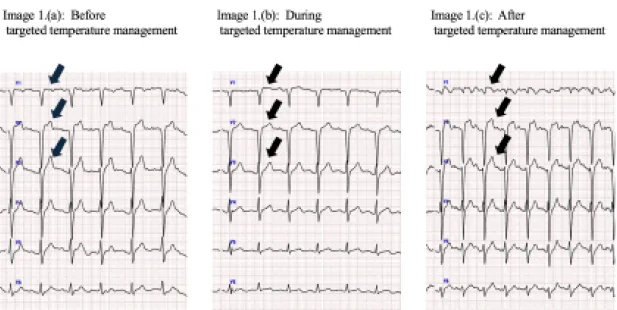
Electrocardiographic changes before, during, and after targeted temperature management in a 44-year-old male with sudden cardiac arrest due to Brugada arrhythmia induced by fever: (a) Before temperature management, showing ST-segment elevation in leads V1–V3 (arrows); (b) During temperature management, showing normal sinus rhythm (arrows); (c) After temperature management, showing a coved-type Brugada electrocardiographic pattern with ST-segment elevation (arrows).
